# Standard diametric versus volumetric early tumor shrinkage as a predictor of survival in metastatic colorectal cancer: subgroup findings of the randomized, open-label phase III trial FIRE-3 / AIO KRK-0306

**DOI:** 10.1007/s00330-022-09053-2

**Published:** 2022-08-17

**Authors:** Felix O. Hofmann, Volker Heinemann, Melvin D’Anastasi, Alena B. Gesenhues, Nina Hesse, Ludwig Fischer von Weikersthal, Thomas Decker, Alexander Kiani, Markus Moehler, Florian Kaiser, Tobias Heintges, Christoph Kahl, Frank Kullmann, Werner Scheithauer, Hartmut Link, Dominik P. Modest, Sebastian Stintzing, Julian W. Holch

**Affiliations:** 1 Department of Radiology, University Hospital, LMU Munich, Marchioninistrasse 15, 81377 Munich, Germany; 2 Department of General, Visceral and Transplantation Surgery, University Hospital, LMU Munich, Marchioninistrasse 15, 81377 Munich, Germany; 3 German Cancer Consortium (DKTK), partner site Munich, and German Cancer Research Centre (DKFZ), Heidelberg, Germany; 4 Department of Medicine III, Comprehensive Cancer Center Munich, University Hospital Grosshadern, LMU Munich, Marchioninistrasse 15, 81377 Munich, Germany; 5 Mater Dei Hospital, University of Malta, Triq tal-Qroqq, Msida, MSD2090 Malta; 6 Klinikum St. Marien Amberg, Amberg, Germany; 7 Onkologische Praxis, Ravensburg, Germany; 8 Department of Medicine IV, Klinikum Bayreuth GmbH, Bayreuth, Germany; 9 Comprehensive Cancer Center Erlangen-EMN (CCC ER-EMN), Erlangen, Germany; 10 Department of Internal Medicine I, University Medical Center Mainz, Mainz, Germany; 11 VK&K Studien GbR, Landshut, Germany; 12 Rheinlandklinikum Neuss, Lukaskrankenhaus, Neuss, Germany; 13 Department of Hematology, Oncology and Palliative Care, Klinikum Magdeburg gGmbH, Magdeburg, Germany; 14 Department of Internal Medicine I, Hospital Weiden, Weiden, Germany; 15 Department of Internal Medicine I and Comprehensive Cancer Center, Medical University of Vienna, Vienna, Austria; 16 Department of Medicine I, Westpfalz-Klinikum GmbH, Kaiserslautern, Germany; 17 Medical Department of Hematology, Oncology and Cancer Immunology (CCM), Charité-Universitätsmedizin Berlin, Berlin, Germany

**Keywords:** Colorectal neoplasms, Neoplasm metastasis, Response Evaluation Criteria in Solid Tumors, Multidetector computed tomography, ROC curve

## Abstract

**Objectives:**

Early tumor shrinkage (ETS) quantifies the objective response at the first assessment during systemic treatment. In metastatic colorectal cancer (mCRC), ETS gains relevance as an early available surrogate for patient survival. The aim of this study was to increase the predictive accuracy of ETS by using semi-automated volumetry instead of standard diametric measurements.

**Methods:**

Diametric and volumetric ETS were retrospectively calculated in 253 mCRC patients who received 5-fluorouracil, leucovorin, and irinotecan (FOLFIRI) combined with either cetuximab or bevacizumab. The association of diametric and volumetric ETS with overall survival (OS) and progression-free survival (PFS) was compared.

**Results:**

Continuous diametric and volumetric ETS predicted survival similarly regarding concordance indices (*p* > .05). In receiver operating characteristics, a volumetric threshold of 45% optimally identified short-term survivors. For patients with volumetric ETS ≥ 45% (vs < 45%), median OS was longer (32.5 vs 19.0 months, *p* < .001) and the risk of death reduced for the first and second year (hazard ratio [HR] = 0.25, *p* < .001, and HR = 0.39, *p* < .001). Patients with ETS ≥ 45% had a reduced risk of progressive disease only for the first 6 months (HR = 0.26, *p* < .001). These survival times and risks were comparable to those of diametric ETS ≥ 20% (vs < 20%).

**Conclusions:**

The accuracy of ETS in predicting survival was not increased by volumetric instead of diametric measurements. Continuous diametric and volumetric ETS similarly predicted survival, regardless of whether patients received cetuximab or bevacizumab. A volumetric ETS threshold of 45% and a diametric ETS threshold of 20% equally identified short-term survivors.

**Key Points:**

*• ETS based on volumetric measurements did not predict survival more accurately than ETS based on standard diametric measurements.*

*• Continuous diametric and volumetric ETS predicted survival similarly in patients receiving FOLFIRI with cetuximab or bevacizumab.*

*• A volumetric ETS threshold of 45% and a diametric ETS threshold of 20% equally identified short-term survivors.*

**Supplementary Information:**

The online version contains supplementary material available at 10.1007/s00330-022-09053-2.

## Introduction

The efficacy of treatment in solid tumors is usually measured by objective response rate (ORR), progression-free survival (PFS), and overall survival (OS). Tumor response and survival times in unresectable metastatic colorectal cancer (mCRC) improved significantly by combining modern chemotherapy with targeted agents [1–3]. Consequently, the direct causality between first-line treatment and survival outcomes is increasingly obliterated by longer follow-up, higher rates of secondary resections, and longer periods of subsequent treatment. Hence, to compare the efficacy of first-line therapeutic regimens, surrogate outcome measurements become more and more important [4, 5].

The Response Evaluation Criteria in Solid Tumors (RECIST) evaluate treatment response based on the assessment of non-target lesions and the diametric measurement of target lesions. However, RECIST’s definition of tumor response is broad and might be too simple to reflect differences within this diverse subgroup adequately. As a result, the quantitative evaluation of tumor shrinkage gains importance as an early available surrogate parameter for patient survival [4].

Early tumor shrinkage (ETS) is defined as the relative reduction of the target lesions’ size from baseline to first restaging. Thus, positive ETS reflects tumor shrinkage during therapy. In most of the previous studies, ETS was calculated based on diametric measurements, and either considered as a continuous variable or categorized using thresholds between 10% [6–8] and 20% [9–12]. In patients with mCRC, ETS proved to be associated with prolonged OS and PFS; however, the predictive values were varying [9, 13, 14]. More consistent measurements might increase ETS’ accuracy.

Volumetric assessments reduce inter- and intra-reader variations [15, 16]. Furthermore, volumetry captures the real size change of lesions that shrink asymmetrically [17]. Therefore, predictions based on volumetric ETS may be more accurate. Also, previous studies showed higher accuracy of volumetric assessments in identifying treatment-sensitive tumors [18]. Considering this within the context of the development of fully automated segmentation algorithms [19], the relevance of the volume of metastases and its change might be of great interest in the coming years.

Therefore, the aim of this subgroup analysis of the FIRE-3 trial [20] was to compare the predictive value of innovative volumetric with standard diametric ETS in the setting of modern combination therapy including either anti-EGFR or anti-VEGF agents.

## Materials and methods

### Patient population

The present study is a retrospective analysis of the FIRE-3 trial. In FIRE-3, patients with histologically confirmed mCRC received 5-fluorouracil, leucovorin, and irinotecan (FOLFIRI) combined with either cetuximab or bevacizumab [20]. The intention-to-treat population included patients with KRAS exon 2 wild-type tumors. Due to the reduced efficacy of EGFR antibodies in patients carrying other RAS mutations, this subgroup should not receive cetuximab. Therefore, reflecting current guidelines [21], this and other analyses of the FIRE-3 trial focused on patients without any RAS mutation [11, 20]. The present study also excluded patients who received less than 30 days first-line therapy.

The study protocol, the full study population, Declaration of Helsinki accordance, and ethical approval were reported earlier [20]. FIRE-3 was registered with ClinicalTrials.gov (NCT00433927). The data cutoff of the current analysis was July 2014. The radiological response has been evaluated earlier [11, 20, 22]; however, none of the previous publications on FIRE-3 reported on volumetric ETS.

### Imaging

The study protocol of FIRE-3 scheduled computed tomographies (CT) at baseline within 2 weeks of the start of therapy, after 6 and 12 weeks of treatment, and from then, every 10 weeks during first-line therapy. As the current study focused on the effect of first-line therapy, only CT conducted after at least 30 days of first-line therapy, but not later than 30 days after its end, were considered.

Due to the multicentric design of the FIRE-3 trial, CT were performed using multidetector computed tomography scanners of various manufacturers at the participating study centers. The weight-adapted dose of the iodinated contrast agent was administered intravenously. Abdominal scans were obtained in portal venous phase, and reconstructed using a standard soft tissue reconstruction kernel with a slice thickness ≤ 5 mm.

### Measurements

The measurements were performed as described earlier [23]: Two radiologists with 4 and 2 years of experience in oncological imaging evaluated the CT at a post-processing workstation using the imaging software syngo.via, MM oncology (VC36, Siemens Healthineers). As prior studies showed a high interobserver agreement for semi-automated volumetric measurements, scans were reviewed by either of them [16].

Up to five evaluable metastases ≥ 1 cm in each of the liver, lungs, and lymph nodes were selected as target lesions. The RECIST 1.1 standard (up to two metastases per organ, five metastases in total) was extended to reduce the influence of nonrepresentative metastases. Consistently with RECIST, large and well-defined metastases were preferred. Borderline cases were discussed with two senior radiologists to reach consensus.

Semi-automated segmentation of the selected target lesions was performed by drawing a diametrical line through the lesion in the axial plane. The software automatically identified all voxels belonging to the lesion by attenuation differences including the neighboring slices. The volumetric segmentation was visually controlled and manually corrected if necessary. Finally, the software calculated the lesion’s largest diameter in the axial plane and its volume. An example of the segmentation is shown in Fig. 2.

### Calculation of ETS

The diameters and the volumes of all segmented metastases were summed up separately. ETS was calculated for both, for the sum of volumes and for the sum of diameters of all measured metastases. It was defined as the relative reduction of the total size from baseline to the next available and eligible examination containing evaluable metastases. Therefore, positive ETS reflects tumor shrinkage.

### Analysis of PFS

In the current study, patients with progressive disease before or at the time of ETS assessment were excluded from analyses regarding the correlation of ETS and PFS. In FIRE-3, progressive disease was prospectively assessed following RECIST 1.0.

### Statistical analysis

Continuous diameter- and volume-based ETS and therapy-specific ETS were compared with the Wilcoxon rank sum test. *p* was adjusted for multiple testing with the Hommel correction.

The hazard rate ratio (HR) for continuous diameter- and volume-based ETS was calculated in Cox proportional hazard regression for OS and PFS. Due to nonlinear associations of ETS and OS, HR depending on ETS was plotted using restricted cubic splines with four knots (at quantiles .05, .35, .65, and .95). Interactions between the treatment arm and ETS were formally tested with Wald statistics.

The prognostic relevance of diameter- and volume-based ETS in predicting OS and PFS was quantified and compared with Harrell’s C concordance index (C index) [24]. The area under the curve (AUC) of the time-dependent receiver operating characteristic (ROC) that identified patients who had died at the time of median OS or had progressive disease at the time of median PFS was calculated.

This ROC was used to determine thresholds for volume- and diameter-based ETS optimally identifying patients with a high risk of death or progressive disease. Ideal cutoffs were selected by maximizing the sum of sensitivity and specificity (Youden index [YI] = sensitivity + specificity − 1) [25]. The thresholds for OS and PFS were adjusted to a uniform cutoff that was easy to remember. The 95% confidence intervals (CI) and *p* values for the differences of sensitivity and specificity were calculated by bootstrapping 1000 times.

Median OS and PFS of patients with ETS below and above the thresholds were determined using the Kaplan-Meier method and compared in log-rank tests. As the assumption of proportional hazard was violated in several cases, time-dependent Cox proportional hazard regression was performed in intervals (OS: 0 to 12, 12 to 24, and > 24 months; PFS: 0 to 6, 6 to 12, and > 12 months).

Finally, the diametric and volumetric thresholds were applied and the proportion of patients achieving ETS compared by the chi-squared test.

Results were considered significant if *p* ≤ .05. All analyses were performed with R version 4.0.3 [26] in RStudio version 1.3.1073. A list of the packages implemented is attached (Data Supplement, Table S1).

## Results

### Patient population

**Fig. 1:**
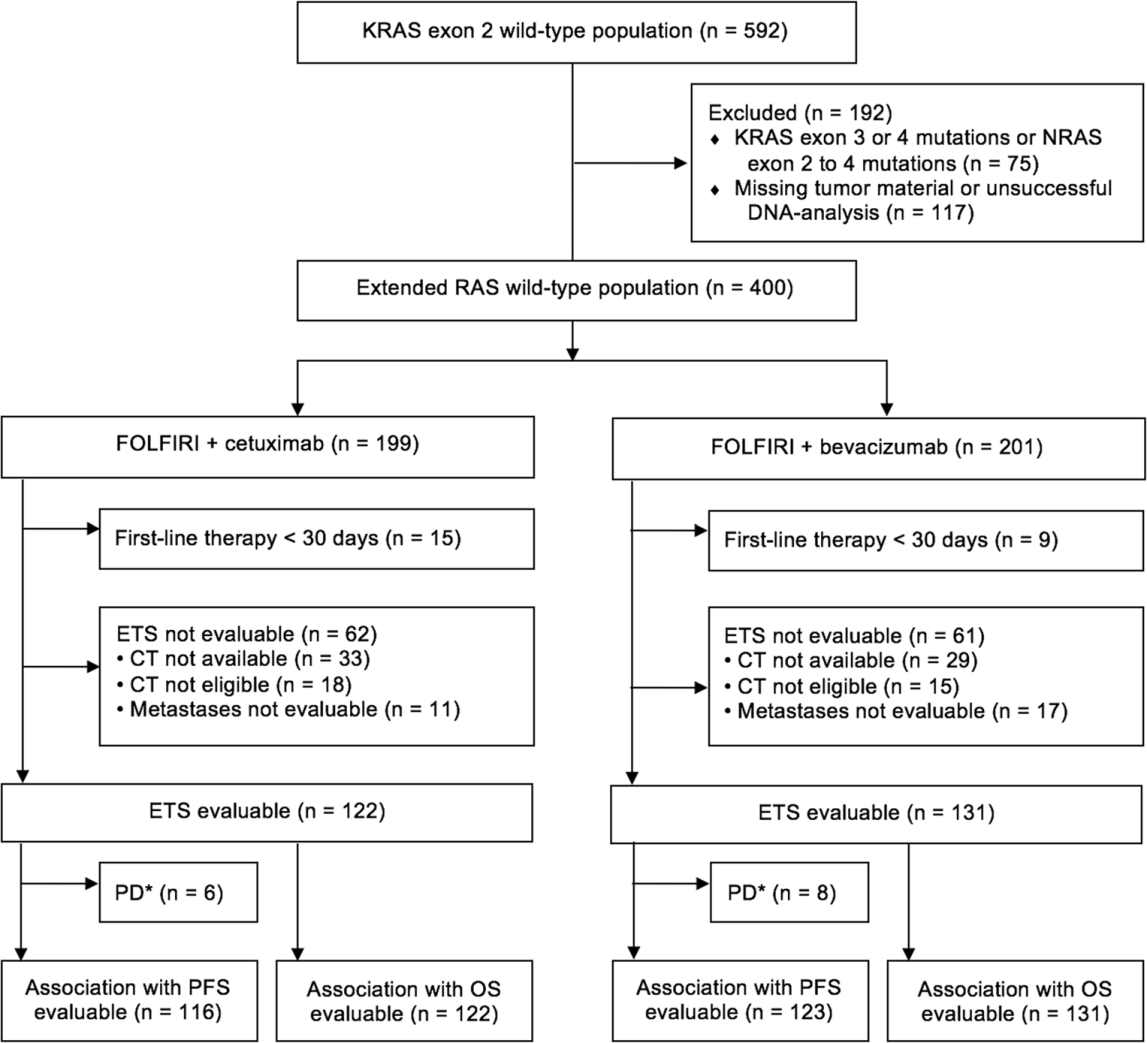
CONSORT diagram. * Patients with progressive disease (PD) before or at the time of ETS assessment were excluded from PFS-related analyses. Abbreviations: CT, computed tomography; n, number of patients; OS, overall survival; PD, progressive disease; PFS, progression-free survival

The intention-to-treat population in FIRE-3 consisted of 592 patients with KRAS exon 2 wild-type tumors (Fig. 1). Four hundred (67.6%) had extended RAS wild-type tumors. We determined ETS in 253 patients with RAS wild-type tumors and available CT imaging. 122/253 (48.2%) of these received FOLFIRI plus cetuximab and 131/253 (51.8%) FOLFIRI plus bevacizumab. Fourteen patients had had progressive disease before or at the time of ETS assessment and were excluded from all analyses regarding PFS.

### Measurements

**Fig. 2:**
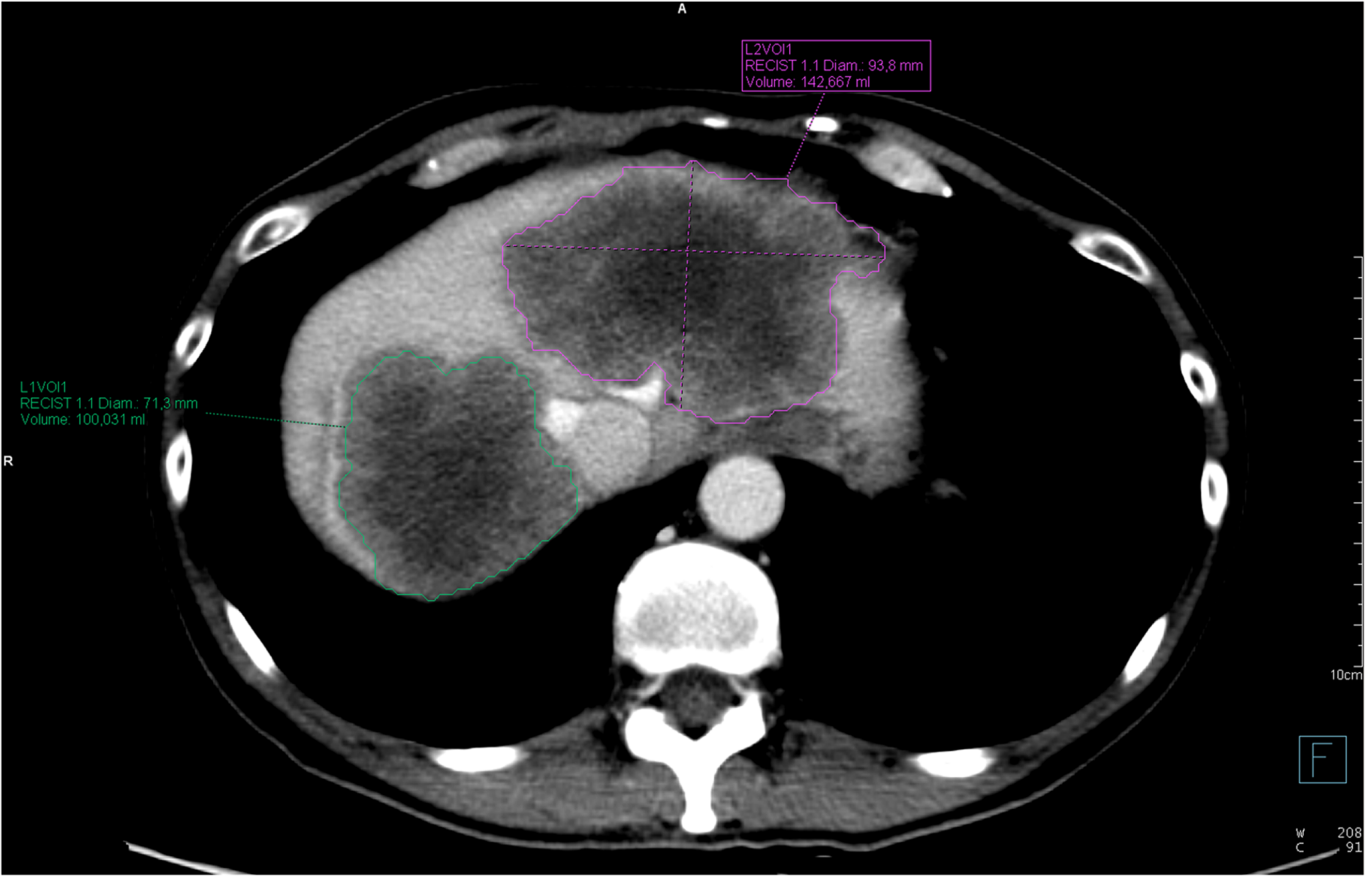
Example of volumetric segmentation. Computed tomography scan of a 52-year-old female at baseline, after segmentation of two large liver lesions as proposed by the software. Afterwards, minor manual corrections of the contours were necessary. One smaller lesion in segment V is not shown. Receiving FOLFIRI plus bevacizumab, the patient had a diametric early tumor shrinkage (ETS) of 26.3% and a volumetric ETS of 65.7% in the first restaging. She had progressive disease after 7.8 months and died after 21.5 months. Abbreviations: ETS, early tumor shrinkage

The CT were performed in median after 6.0 weeks (inter-quartile range [IQR], 5.4-7.1 weeks) of therapy. We measured the diameters and volumes of 1005 metastases, an average of 4.0 (standard deviation [SD], 2.0) per patient. Their diameters and volumes at baseline measured in median 28.3 mm (IQR, 18.3–45.2 mm) and 7.9 cm^3^ (IQR, 2.6–26.7 cm^3^), respectively. Figure 2 shows a representative example of the segmentation. In the current cohort, median OS was 26.4 months (95% CI, 23.7–30.8) and median PFS 10.8 months (95% CI, 10.2–12.1).

Obviously, ETS (defined as relative reduction of size; therefore, positive ETS reflects tumor shrinkage) was significantly larger if calculated based on volume rather than diameter measurements (median 54% [IQR, 33–73] vs 23% [13–36], *p* < .001; Supplement, Fig. S1).

### Continuous ETS

**Fig. 3:**
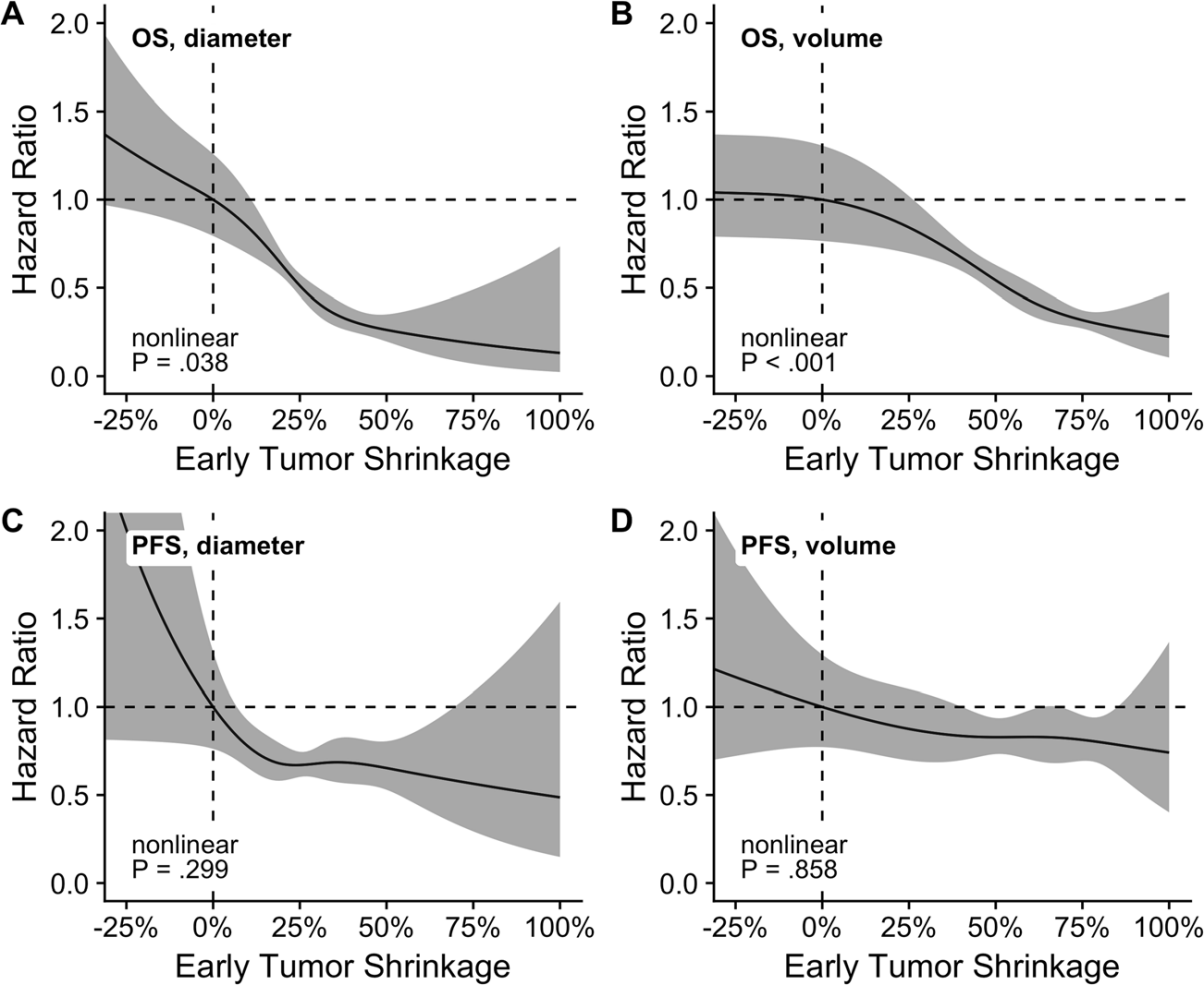
Risk of death or progressive disease. Hazard rate ratio and its 95% confidence interval (CI) of the risk to die (**A**, **B**) or to have progressive disease (**C**, **D**) depending on diametric (**A**, **C**) and volumetric (**B**, **D**) early tumor shrinkage between − 25 and 100%. Plotted using restricted cubic splines with four knots at the quantiles .05, .35, .65, and .95. Abbreviations: OS, overall survival; PFS, progression-free survival

ETS treated as a continuous, non-binary variable was nonlinearly associated with OS, and linearly associated with PFS in Cox proportional hazard regression (OS: diameter: *p* = .038, volume: *p* < .001; PFS: diameter: *p* = .299; volume: *p* = .858). Therefore, from a statistical point of view, it would not be appropriate to calculate specific hazard ratios for the association between ETS and OS. The visual analysis performed instead shows that higher ETS correlates clearly with a reduced risk of dying (Fig. 3A, B) and a reduced risk of progressive disease (Fig. 3C, D).

The effect of diametric and volumetric ETS on OS and PFS was independent of the treatment in interaction tests (OS: diametric ETS *p* = .876, volumetric ETS *p* = .340; PFS: diametric ETS *p* = .393, volumetric ETS *p* = .207). Sliding windows in subpopulation treatment effect pattern plots (STEPP) confirmed this (Supplement, Fig. S2). We therefore considered a uniform ETS threshold for bevacizumab and cetuximab as reasonable.

**Fig. 4:**
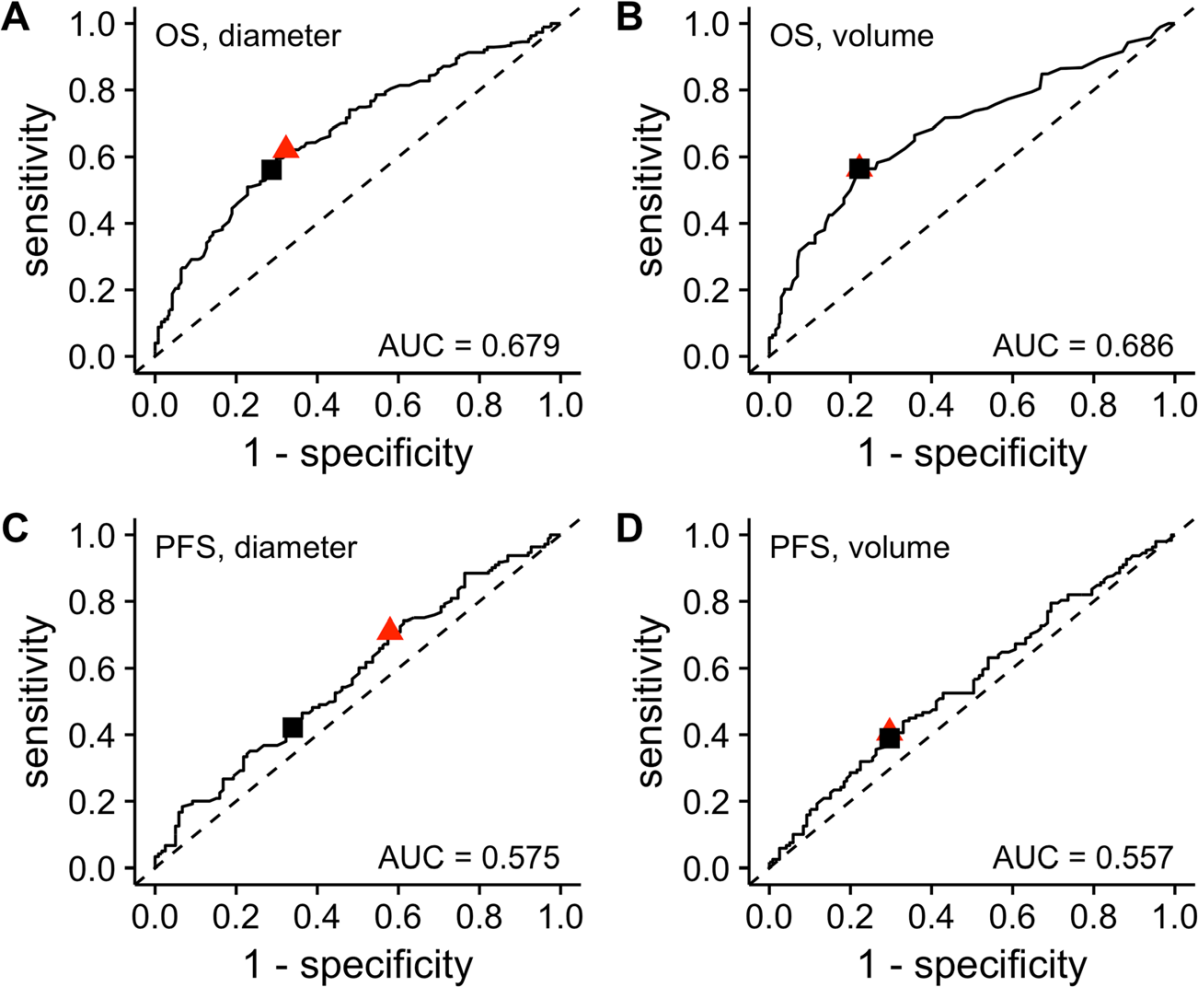
Time-dependent receiver operating characteristics. Time-dependent receiver operating characteristics at median OS (26.4 months) for diameter- (**A**) and volume-based (**B**) early tumor shrinkage and at median PFS (10.8 months) for diameter- (**C**) and volume-based (**D**) early tumor shrinkage. The red triangle highlights the point of the maximal sum of sensitivity and specificity, the black square the point of the chosen threshold. Abbreviations: AUC, area under the curve; OS, overall survival; PFS, progression-free survival

Volume- and diameter-based ETS predicted OS and PFS comparably: C indices did not differ significantly (OS: *p* = .780; PFS: *p* = .259; Supplement, Table S2) and the AUC of ROC were similar (Fig. 4). Higher C indices and larger AUC indicated that volume- and diameter-based ETS predicted OS more accurately than PFS in all analyses. In summary, the performance of volume-based ETS and that of diameter-based ETS in predicting OS and PFS were comparable, and better for OS than for PFS.

### Optimization of ETS thresholds

To categorize patients into responders and non-responders, cutoffs are necessary. We identified thresholds optimally predicting death or progressive disease by maximizing the sum of sensitivity and specificity (Youden index, YI) in ROC analyses. For OS, we determined a diametric threshold of 21.4% and a volumetric threshold of 45.4%. For PFS, the diametric threshold was 33.2% and the volumetric threshold, 45.3% (Fig. 4; Supplement, Tables S3, S4).

The optimized diametric and volumetric ETS thresholds did not significantly differ in predicting death or progressive disease in general (YI, OS: *p* = .350; YI, PFS: *p* = .786), but in separate analysis of sensitivity and specificity (Supplement, Tables S5, S6). OS was predicted more accurately than PFS, especially by the volumetric threshold (*p* = .002) (Supplement, Tables S7, S8).

### Performance of proposed ETS thresholds

These thresholds are optimized regarding sensitivity and specificity and may be specific to the underlying dataset. For diametric ETS, the threshold of ≥ 20% is well established [9–12] and was mostly consistent with our findings. For volumetric ETS, we proposed a uniform threshold of ≥ 45%, being a simplification of the optimized thresholds for predicting OS (45.4%) and PFS (45.3%). The established diametric and the proposed volumetric ETS thresholds did not differ regarding sensitivity (OS: *p* = .887; PFS: *p* = .325), specificity (OS: *p* = .097; PFS: *p* = .236), and YI (OS: *p* = .143; PFS: *p* = .826) (Supplement, Tables S9, S10).

### Survival of responders vs non-responders

**Fig. 5:**
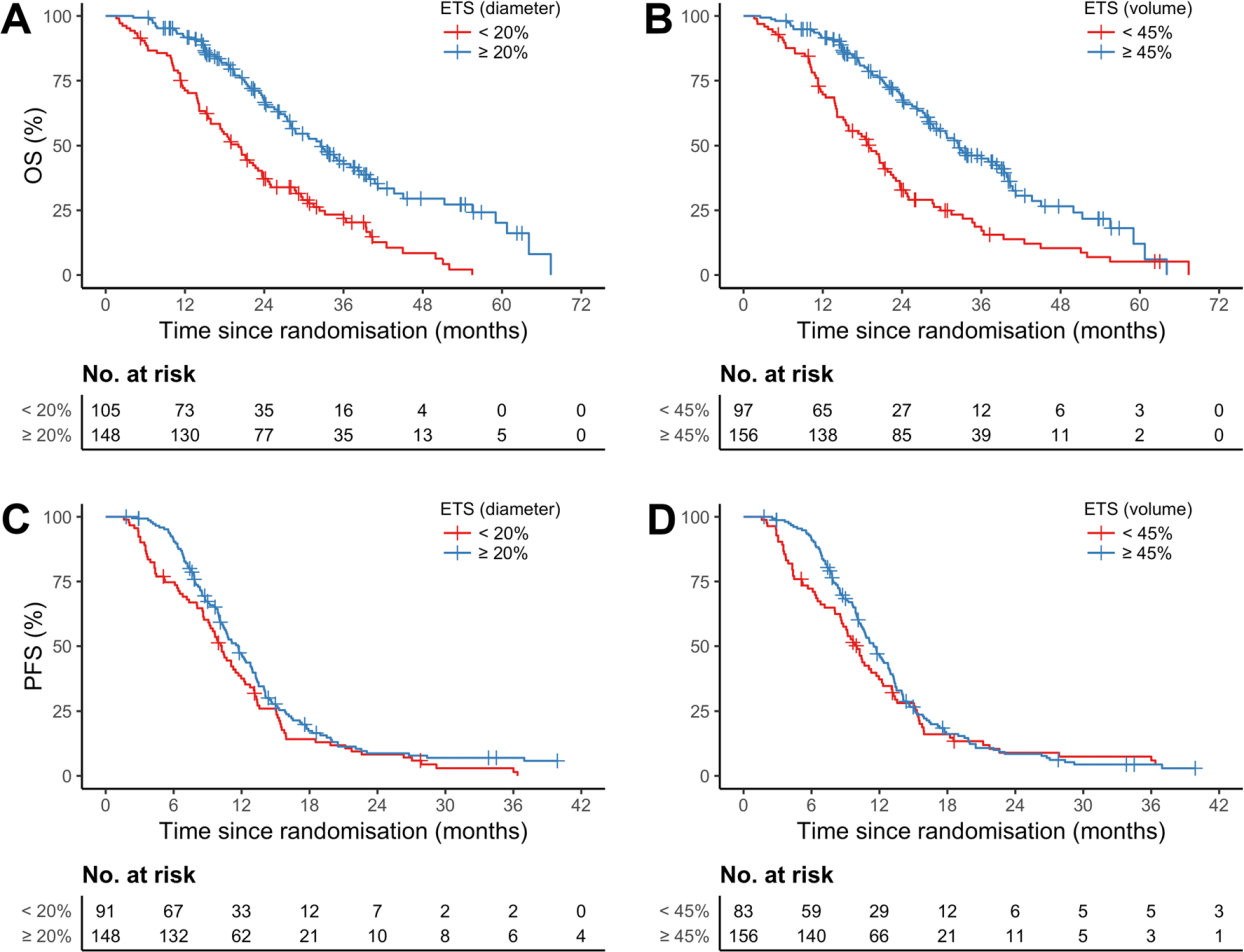
Kaplan-Meier survival curves. Kaplan-Meier survival curves for OS (**A**, **B**) and PFS (**C**, **D**) comparing patients with and without diametric (**A**, **C**), respectively volumetric (**B**, **D**) ETS. Abbreviations: ETS, early tumor shrinkage; No., number of patients; OS, overall survival; PFS, progression-free survival

Both the diametric and the volumetric thresholds separated short- and long-term survivors (Table 1, Fig. 5), and median OS times were similarly prolonged for patients achieving diametric and volumetric ETS (32.5 vs 20.1 months, *p* < .001, and 32.5 vs 19.0 months, *p* < .001, respectively). Separate analyses of the treatment arms confirmed these findings (Supplement, Fig S3). Survival curves and times comparing the newly proposed thresholds with the established RECIST thresholds are shown in the supplement (Supplement, Fig S4).

**Table 1:** Overall survival and progression-free survival of patients with diametric and volumetric ETS below and above the specific threshold, determined in Kaplan-Meier analysis. Groups were compared with log-rank test

Outcome	Diameter-based ETS		Volume-based ETS	
	< 20%	≥ 20%	< 45%	≥ 45%
Median OS, months [95% CI]	20.1 [15.9 to 23.7]	32.5 [28.0 to 38.7]	19.0 [15.4 to 22.7]	32.5 [28.7 to 39.9]
	*p* < .001		*p* < .001	
Median PFS, months [95% CI]	10.3 [9.1 to 12.0]	11.7 [10.4 to 12.9]	10.0 [8.7 to 12.0]	11.5 [10.4 to 12.9]
	*p* = .056		*p* = .381	

*CI* confidence interval, *ETS* early tumor shrinkage, *n* number of patients

ETS was highly associated with OS in time-dependent Cox proportional hazard regression (Table 2): For the first and second year, the risk of dying was significantly reduced for patients achieving diametric or volumetric ETS. After 2 years, only patients who had achieved diametric ETS had a significant risk reduction. Formally tested, the treatment did not influence the effect of ETS on OS in time-dependent analyses (all *p* > .20).

**Table 2:** Time-dependent Cox proportional hazard regression determining the risk associated with diametric and volumetric ETS above the specific threshold depending on the time

Outcome	Diameter-based ETS	Volume-based ETS
Time interval	< 20% vs ≥ 20% HR [95% CI]	< 45% vs ≥ 45% HR [95% CI]
Overall survival		
0–12 months	0.25 [0.13 to 0.49], *p* < .001	0.25 [0.13 to 0.47], *p* < .001
12–24 months	0.47 [0.29 to 0.76], *p* = .002	0.39 [0.24 to 0.64], *p* < .001
> 24 months	0.56 [0.33 to 0.95], *p* = .033	0.87 [0.50 to 1.52], *p* = .622
Progression-free survival		
0–6 months	0.30 [0.15 to 0.60], *p* < .001	0.26 [0.13 to 0.52], *p* < .001
6–12 months	1.01 [0.66 to 1.54], *p* = .966	1.05 [0.67 to 1.63], *p* = .843
> 12 months	0.85 [0.54 to 1.34], *p* = .486	1.31 [0.81 to 2.12], *p* = .267

*CI* confidence interval, *ETS* early tumor shrinkage, *HR* hazard rate ratio, *n* number of patients

The predictive value of ETS for PFS was insignificant (Table 1, Fig. 5), and median PFS times were not prolonged for patients achieving diametric (11.7 vs 10.3 months, *p* = .056) or volumetric ETS (11.5 vs 10.0 months, *p* = .381). In time-dependent Cox proportional hazard regression, the risk of progressive disease was significantly reduced only during the first 6 months, but not afterwards (Table 2).

### Diametric vs volumetric categorization

Diametric ETS ≥ 20% was observed in 148/253 (58.5%) and volumetric ETS ≥ 45% in 156/253 (61.7%) patients (*p* = .525). Independent of the method, we consistently classified 136 (53.8%) as achieving and 85 patients (33.6%) as not achieving ETS. Twenty patients (7.9%) only achieved volumetric ETS and 12 patients (4.7%) only diametric ETS.

### Secondary resectability

Diametric and volumetric ETS were both associated with secondary resectability of metastases: Metastases were resected in 40/148 (27.0%) of the patients achieving diametric ETS ≥ 20%, compared to 13/105 (12.4%) of the patients with diametric ETS < 20% (*p* = .006). Likewise, metastases were resected in 41/156 (26.3%) of the patients achieving volumetric ETS ≥ 45%, compared to 12/97 (12.4%) of the patients with volumetric ETS < 45% (*p* = .011).

## Discussion

Early tumor shrinkage (ETS) is defined as tumor shrinkage from baseline to the first restaging. In metastatic colorectal cancer (mCRC), it is associated with survival and offers additional value by evaluating the efficacy of first-line systemic therapy independently from the subsequent treatment. Our hypothesis was that innovative volumetric ETS represents size changes of metastases more accurately than standard diametric ETS and is consequently more reliable as surrogate outcome.

In this study, however, volumetric ETS did not differ from standard diametric ETS in predicting survival in RAS wildtype mCRC despite promising results in previous studies [18, 27]. Volumetric and diametric ETS treated as continuous variables were equally associated with survival outcomes. In addition, after categorization of patients into responders and non-responders, volumetric ETS ≥ 45% and diametric ETS ≥ 20% predicted OS and PFS equally. Thus, diametric measurements might already represent size changes adequately and uncovered asymmetric size changes might be in average not of major importance. If volumetry is performed nevertheless, for instance to further evaluate texture of metastases [28], the usage of volumetric ETS appears in the light of the current study feasible as well.

Altogether, our findings strengthen previous efforts to implement diametric ETS as the key secondary endpoint in prospective clinical trials [29, 30]*.* The prospective evaluation of ETS and its integration into adaptive trial designs are crucial steps toward its implementation into routine clinical practice.

The distinction between responders and non-responders requires the definition of a threshold. By maximizing sensitivity and specificity in receiver operating characteristics, we determined thresholds that optimally identify patients surviving shorter than median. For diametric ETS, the threshold optimized to predict OS was 21.4%, confirming the established diametric cutoff at 20%. The proportion of patients achieving diametric ETS ≥ 20% and corresponding survival times were mainly comparable to those of other studies with corresponding treatment arms [9, 11, 12]. For volumetric ETS, the threshold optimized to predict OS was 45.4%, simplified to 45%.

It was of note that the choice of targeted therapy (anti-EGFR vs anti-VEGF) did not influence the effect of ETS on OS or PFS significantly. This is in line with previous evaluations [31] and suggests that the broadly accepted diametric ETS threshold at 20% as well as our proposed volumetric ETS threshold at 45% might be applicable independently of the targeted agent.

Early tumor shrinkage (ETS) is supposed to measure the sensitivity to treatment as early as possible, and therefore quantifies the tumor shrinkage at the earliest restaging. RECIST instead categorizes patients into responders or non-responders at any time, covering the whole course of disease. These different purposes and definitions might drive advantages of ETS over classical response rate according to RECIST [4] but hamper comparability and cause different thresholds. The ETS thresholds defined in this and in other studies are less conservative than the RECIST thresholds. For diametric ETS, a shrinkage of 20% was considered relevant, compared to 30% according to RECIST. For volumetric ETS, we defined 45% as significant, compared to 65% derived from RECIST [23]. These differences are in parts caused by the timing of the CT scan: Tumor shrinkage at the first restaging as measured by ETS might be smaller by definition than the maximal tumor response over the whole course of disease as measured by RECIST. In other words, patients who are classified as responders according to ETS at the first restaging might be non-responders according to RECIST at this time but become responders according to RECIST at the second or third restaging.

The associated risk reduction for both diametric and volumetric ETS decreased over time: Patients achieving ETS had a significantly reduced risk of death for 2 years and a significantly reduced risk of progressive disease for 6 months. Afterwards, the risk of death or progress was comparable to patients not achieving ETS. This must be interpreted with caution due to the retrospective selection of the regressions’ time intervals and might partially be caused by the determination of the thresholds in ROC analyses at median survival. Nevertheless, ETS seems to be more accurate in predicting death or progressive disease in the short term, which might be relevant for future studies.

Different biological rationales explain the association between tumor shrinkage as measured by ETS and survival. First, tumor shrinkage represents the sensitivity to systemic treatment. Morphological response to therapy is a surrogate for the destruction of malignant cells and associated with prolonged survival in general. Furthermore, and second, findings of Palmieri et al indicate that response to first-line chemotherapy is also associated with response to subsequent treatment lines [32]. Therefore, high ETS might identify patients whose disease is generally approachable by systemic treatment. Third, in oligometastatic mCRC, the goal of systemic treatment should be secondary resectability that prolongs survival significantly [33]. High ETS can identify patients that are more likely to be converted to secondary resectability [34]. The current study confirmed this, and metastases were more frequently resected in patients who achieved ETS, regardless of whether determined by diametric or volumetric measurement.

Of note, we found no significant association between ETS and PFS, contrary to other studies [4, 13, 14] and an earlier analysis of FIRE-3 [11]. This can be partly explained as this retrospective analysis included less patients than earlier analyses due to more stringent inclusion criteria. For example, we excluded patients who had progressive disease at the time of ETS assessment. Furthermore, PFS might not be an adequate surrogate endpoint for OS in mCRC as post-progressive survival becomes longer, subsequent therapies more effective and its influence on OS more relevant [5, 32]. However, our graphical assessment showed an association of ETS with reduced risk of progressive disease, and we therefore also performed all analyses for PFS.

Our study should be interpreted in the context of its design. Firstly, selected target lesions might not represent the tumor burden properly. However, this does not limit the comparison of diameter- and volume-based ETS, and the measurement of up to five metastases in each of the liver, lungs, and lymph nodes should be a good approximation. Secondly, we determined the thresholds based on the survival at a specific time point. Nevertheless, subsequently, we analyzed the thresholds’ implications on survival in general with the Kaplan-Meier analysis and time-dependent Cox proportional hazard regression. Thirdly, ETS is generally hampered by a possible bias due to the exclusion of patients who have died, been progressive, or had severe adverse effects before the first CT after baseline. Finally, the current study is retrospective and was not upfront powered for the conducted analyses.

Altogether, high ETS represents the sensitivity to systemic treatment at the earliest restaging and was associated with prolonged survival. Volumetric instead of standard diametric measurements did not increase the predictive accuracy of ETS in the current study. The confirmed diametric ETS threshold at 20% and our proposed volumetric ETS threshold at 45% equally identified short-term survivors, independently of the treatment. These findings strengthen previous efforts to validate diametric ETS as surrogate parameter for overall survival and pave the way to its implementation in clinical routine.

## Supplementary Information

ESM 1 (PDF 1114 kb)

## Abbreviations

AUC: Area under the curve
CI: Confidence interval
C index: Harrel’s C concordance index
CT: Computed tomography
ETS: Early tumor shrinkage
FOLFIRI: 5-Fluorouracil, leucovorin, and irinotecan
HR: Hazard rate ratio
IQR: Inter-quartile range
mCRC: Metastatic colorectal cancer
OS: Overall survival
PFS: Progression-free survival
RECIST: Response Evaluation Criteria in Solid Tumors
ROC: Receiver operating characteristic
SD: Standard deviation
STEPP: Subpopulation treatment effect pattern plot

## Declarations

### Guarantor

The scientific guarantor of this publication is Felix O. Hofmann.

### Statistics and biometry

One of the authors has significant statistical expertise.

### Study subjects or cohorts overlap

- Heinemann et al, Lancet Oncol 2014; 15(10): 1065-75
- Stintzing et al, Ann Oncol 2014; 25(supp5): 1-41
- Stintzing et al, Lancet Oncol 2016; 17(10):1426-34
- Winter/Hofmann et al, Eur Radiol 2018; 28(11): 4839-48

None of our previous publications or conference contributions reported on volumetric ETS of the FIRE-3 population.

### Methodology

• Retrospective

• diagnostic or prognostic

• multicenter study
