# Supplementary material for: Standard diametric versus volumetric early tumor shrinkage as a predictor of survival in metastatic colorectal cancer: subgroup findings of the randomized, open-label phase III trial FIRE-3 / AIO KRK-0306
Source: Eur Radiol. 2022 Aug 17;33(2):1174–84. doi: 10.1007/s00330-022-09053-2 (PMC9889429; doi:10.1007/s00330-022-09053-2)
Supplement: Supplementary file 1 — (PDF 1114 kb) [file 330_2022_9053_MOESM1_ESM.pdf]

Data Supplement

DS Fig S1

Distribution of early tumor shrinkage, depending on diameter- (red) or volume-based (blue) calculation. Horizontal line shows median early tumor shrinkage [inter-quartile range]. Distributions were significantly different in Wilcoxon rank sum test (*P* < .001).

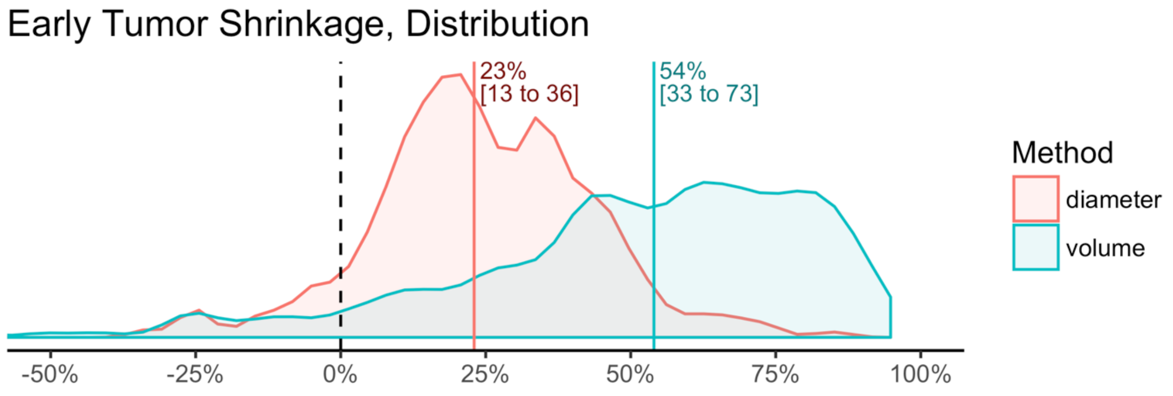

Abbreviations: ETS, early tumor shrinkage.

DS Fig S2

Subpopulation treatment effect pattern plots (STEPP, up to 50 patients per subpopulation, 30 patients overlapping) compare bevacizumab and cetuximab regarding the proportion of patients living at the time of median OS (A, B), respectively the proportion of patients being progression-free at the time of median PFS (E, F). Hazard ratio plots compare the risk of dying (C, D) respectively having tumor progress (G, H) between treatment arms. HR > 1 indicates that patients had a better prognosis if achieving the respective ETS under cetuximab-treatment. All plots depending on subpopulation groups of different ETS, calculated based on diametric (A, C, E, G), respectively volumetric (B, D, F, H) measurements.

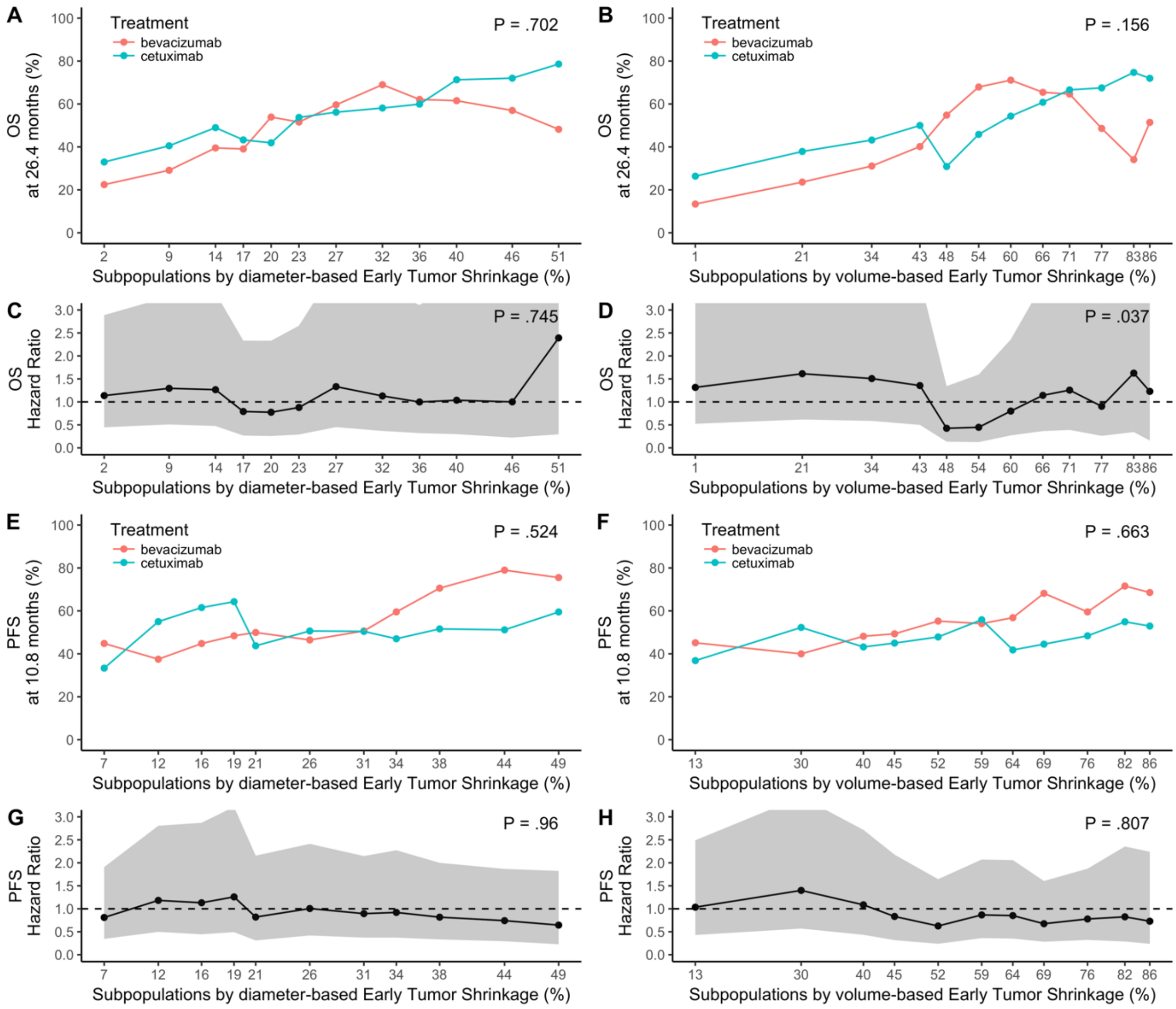

Abbreviations: ETS, early tumor shrinkage; HR, hazard rate ratio; OS, overall survival; PFS, progression-free survival.

DS Fig S3

Kaplan Meier survival curves for overall survival (A, B) and progression-free survival (C, D) comparing patients with and without diametric (A, C), respectively volumetric (B, D) early tumor shrinkage. Additional separation regarding treatment arms.

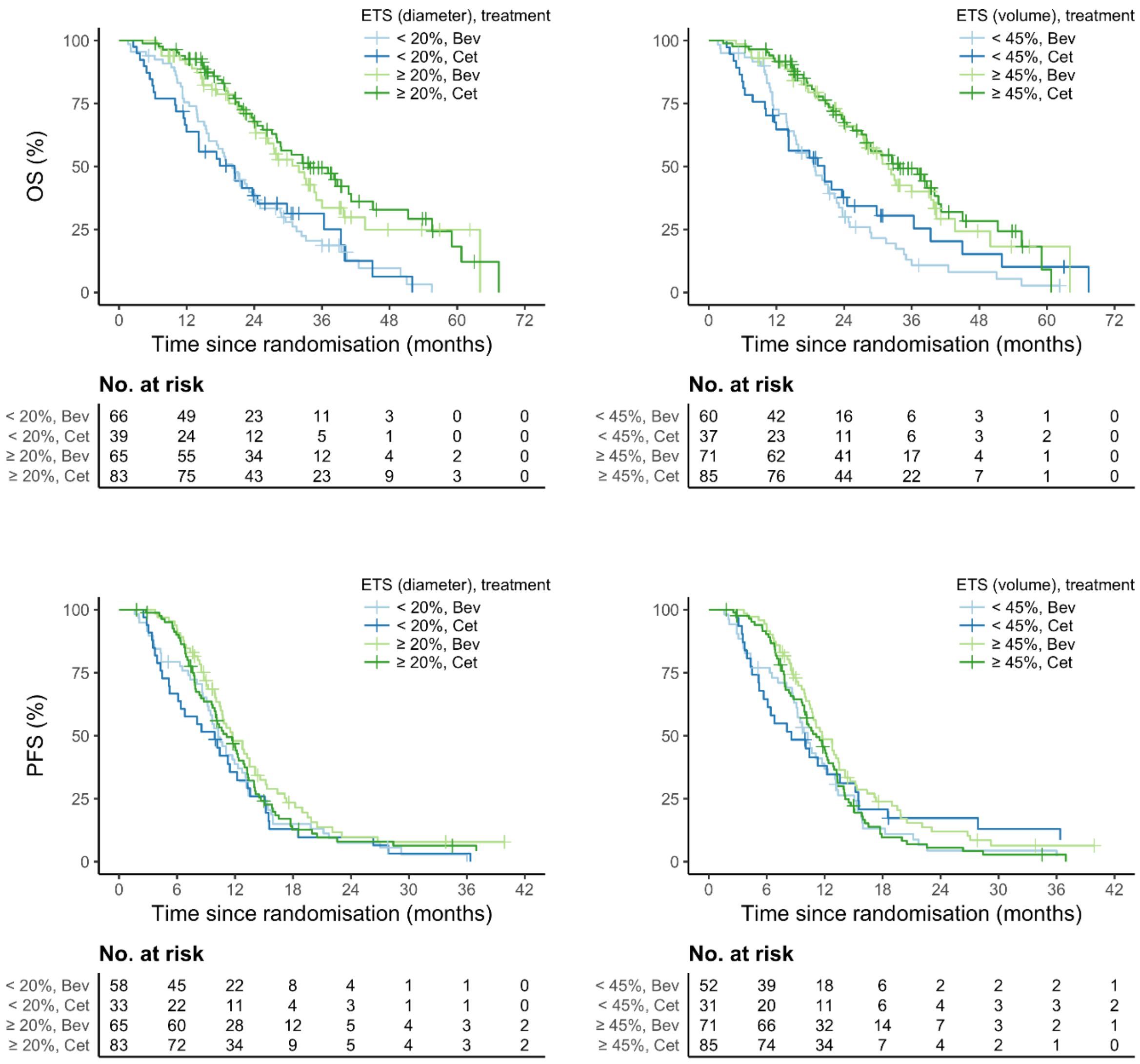

Abbreviations: Bev, bevacizumab; Cet, cetuximab; ETS, early tumor shrinkage; No., number of patients; OS, overall survival; PFS, progression-free survival.

DS Fig S3, Corresponding survival times

Outcome		diameter-based ETS		volume-based ETS	
Treatment		< 20%	≥ 20%	< 45%	≥ 45%
**Overall survival**					
bevacizumab (n = 133)	median OS, m<br>[95%-CI]	20.1<br>[15.9 to 24.8]	31.9<br>[26.1 to 39.9]	18.6<br>[15.4 to 23.1]	31.9<br>[27.4 to 40.4]
		P < .001		*P* < .001	
cetuximab (n = 122)	median OS, m<br>[95%-CI]	20.5<br>[14.1 to 36.4]	33.8<br>[28.0 to 45.0]	20.5<br>[14.1 to 36.4]	33.8<br>[28.0 to 41.2]
		P < .001		*P* = .009	
**Progression-free survival**					
bevacizumab (n = 123)	median PFS, m<br>[95%-CI]	10.3<br>[9.3 to 13.1]	11.7<br>[10.5 to 14.1]	10.3<br>[9.1 to 13.1]	11.8<br>[10.7 to 14.1]
		P = .116		*P* = .085	
cetuximab (n = 116)	median PFS, m<br>[95%-CI]	9.9<br>[6.1 to 13.6]	11.1<br>[9.9 to 13.0]	8.5<br>[5.7 to 15.2]	11.1<br>[9.9 to 13.0]
		P = .181		*P* = .697	

Abbreviations: ETS, early tumor shrinkage; m, months; OS, overall survival; PFS, progression-free survival.

DS Fig S4

Kaplan Meier survival curves for overall survival (A, B) and progression-free survival (C, D) comparing patients categorized as non-responders according to RECIST and ETS, as responders according to RECIST and ETS, or inconsistently as responders according to ETS but non-responders according to RECIST based on diametric (A, C), respectively volumetric (B, D) measurements.

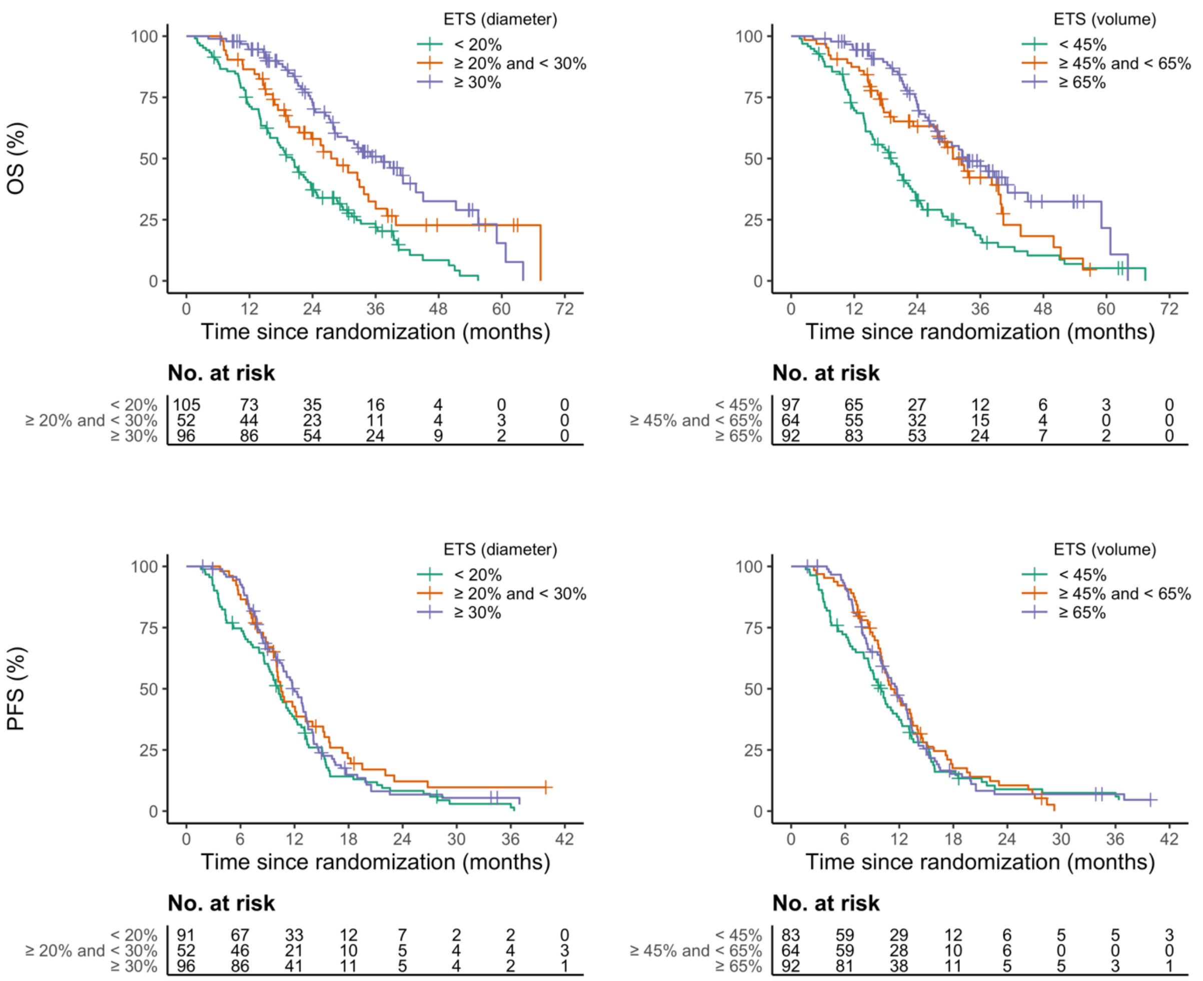

Abbreviations: ETS, early tumor shrinkage; No., number of patients; OS, overall survival; PFS, progression-free survival.

DS Fig S4, Corresponding survival times

Outcome	diameter- based ETS			volume-based ETS		
	< 20%	in between	≥ 30%	< 45%	in between	≥ 65%
**median OS, m [95%-CI]**	20.1 [15.9 to 23.7]	27.5 [21.5 to 36.0] *P < .001*	37.1 [28.7 to 51.3]	19.0 [15.4 to 22.7]	30.8 [27.5 to 40.0] *P < .001*	33.2 [28.0 to 45.0]
**median PFS, m [95%-CI]**	10.3 [9.1 to 12.0]	10.5 [10.0 to 15.2] *P = .100*	12.0 [10.7 to 13.3]	10.0 [8.7 to 12.0]	11.1 [10.2 to 13.5] *P = .700*	11.7 [10.1 to 13.0]

Abbreviations: ETS, early tumor shrinkage; m, months; OS, overall survival; PFS, progression-free survival.

DS Table S1

Packages used for statistical analyses within statistical software R version 4.0.3 - "Bunny-Wunnies Freak Out" (2020-10-10) in RStudio version 1.3.1073 "Giant Goldenrod" (2020-07-29).

Name	Version	Purpose
plyr	1.8.6	Data processing
reshape2	1.4.4	Data processing
data.table	1.13.6	Data processing
tdROC	1.0	Time-dependent ROC-analysis
rms	6.1-0	Cox proportional hazard regression
survival	3.2-7	Survival analysis
compareC	1.3.1	Comparison of correlated C-indices
ggplot2	3.3.3	Image & plot creation
gridExtra	2.3	Plot arrangement
cowplot	1.1.1	Plot arrangement

DS Table S2

Harrel's C concordance-index (C-index) describing the accuracy of diameter- and volume-based ETS in predicting overall survival and progression-free survival, for both study arms together and separately. Comparison and calculation of *P* according to Kang et al.[1].

End point	Treatment	diameter-based ETS		volume-based ETS
Overall survival	both arms (n = 253)	0.659	*P* = .780	0.656
	bevacizumab (n = 131)	0.628	*P* = .946	0.627
	cetuximab (n = 122)	0.684	*P* = .989	0.684
Progression-free survival	both arms (n = 239)	0.555	*P* = .259	0.544
	bevacizumab (n = 123)	0.566	*P* = .827	0.563
	cetuximab (n = 116)	0.557	*P* = .625	0.548

Abbreviations: ETS, early tumor shrinkage.

1. Kang L, Chen W, Petrick NA, et al: Comparing two correlated C indices with right-censored survival outcome: a one-shot nonparametric approach. Stat Med 34:685-703, 2015

DS Table S3

Optimized and established thresholds for the prediction of overall survival and progression-free survival for diameter- and volume-based ETS. Table shows additionally the corresponding sensitivity and specificity within both study arms together, within the bevacizumab- and within the cetuximab-arm.

End point	Treatment		diameter-based ETS		volume-based ETS	
			optimized	established	optimized	proposed
Overall survival		threshold	21.4	20	45.4	45
	both arms (n = 253)	sensitivity	0.621	0.561	0.564	0.564
		specificity	0.677	0.713	0.778	0.778
	Bevacizumab (n = 133)	sensitivity	0.713	0.619	0.645	0.631
		specificity	0.541	0.614	0.725	0.741
	Cetuximab (n = 122)	sensitivity	0.495	0.458	0.464	0.464
		specificity	0.776	0.791	0.811	0.811
Progression-free survival		threshold	33.2	20	45.3	45
	both arms (n = 239)	sensitivity	0.709	0.422	0.406	0.389
		specificity	0.421	0.66	0.703	0.703
	Bevacizumab (n = 123)	sensitivity	0.862	0.528	0.499	0.499
		specificity	0.311	0.566	0.649	0.649
	Cetuximab (n = 116)	sensitivity	0.552	0.314	0.303	0.303
		specificity	0.539	0.748	0.771	0.771

Abbreviations: ETS, early tumor shrinkage; n: number of patients.

DS Table S4

Optimized and established / proposed diametric and volumetric ETS-thresholds for the prediction of death and progressive disease with corresponding sensitivity, specificity and YI. 95%-CI were obtained from bootstrapping 1000 times.

End point		diameter-based ETS		volume-based ETS	
		optimized	established	optimized	proposed
Overall survival	threshold	21.4	20	45.4	45
	sensitivity	0.621	0.561	0.564	0.564
	[95%-CI]	[0.512 to 0.714]	[0.456 to 0.647]	[0.459 to 0.654]	[0.451 to 0.645]
	specificity	0.677	0.713	0.778	0.778
	[95%-CI]	[0.578 to 0.753]	[0.622 to 0.789]	[0.688 to 0.843]	[0.688 to 0.844]
	YI	0.298	0.274	0.342	0.342
	[95%-CI]	[0.134 to 0.415]	[0.129 to 0.398]	[0.198 to 0.449]	[0.19 to 0.444]
Progression-free survival	threshold	33.2	20	45.3	45
	sensitivity	0.709	0.422	0.406	0.389
	[95%-CI]	[0.623 to 0.792]	[0.339 to 0.516]	[0.314 to 0.496]	[0.304 to 0.489]
	specificity	0.421	0.66	0.703	0.703
	[95%-CI]	[0.333 to 0.509]	[0.573 to 0.751]	[0.617 to 0.787]	[0.617 to 0.788]
	YI	0.13	0.082	0.109	0.093
	[95%-CI]	[0.008 to 0.252]	[-0.035 to 0.207]	[-0.017 to 0.232]	[-0.024 to 0.222]

Abbreviations: CI, confidence interval; ETS, early tumor shrinkage; YI, Youden Index.

DS Table S5

Diametric *v* volumetric ETS: Comparison of sensitivity, specificity and YI of optimized diametric threshold predicting death before 26.4 months and optimized volumetric threshold predicting death before 26.4 months after therapy start. 95%-CI and *P* were obtained from bootstrapping 1000 times.

End point<br>Measurement<br>Method<br>threshold	OS<br>diameter<br>optimized<br>21.4	OS<br>volume<br>optimized<br>45.4	Difference	*P*
sensitivity	0.621	0.564	0.056	.045
[95%-CI]	[0.512 to 0.714]	[0.459 to 0.654]	[0.006 to 0.114]	
specificity	0.677	0.778	-0.101	.013
[95%-CI]	[0.578 to 0.753]	[0.688 to 0.843]	[-0.18 to -0.035]	
YI	0.298	0.342	-0.044	.350
[95%-CI]	[0.134 to 0.415]	[0.198 to 0.449]	[-0.14 to 0.047]	

Abbreviations: CI, confidence interval; OS, overall survival; YI, Youden Index.

DS Table S6

Diametric *v* volumetric ETS: Comparison of sensitivity, specificity and YI of optimized diametric threshold predicting progressive disease before 10.8 months and optimized volumetric threshold predicting progressive disease before 10.8 months after therapy start. 95%-CI and *P* were obtained from bootstrapping 1000 times.

End point<br>Measurement<br>Method<br>threshold	PFS<br>diameter<br>optimized<br>33.2	PFS<br>volume<br>optimized<br>45.3	Difference	*P*
sensitivity<br>[95%-CI]	0.709<br>[0.623 to 0.792]	0.406<br>[0.314 to 0.496]	0.303<br>[0.224 to 0.399]	< .001
specificity<br>[95%-CI]	0.421<br>[0.333 to 0.509]	0.703<br>[0.617 to 0.787]	-0.283<br>[-0.365 to -0.201]	< .001
YI<br>[95%-CI]	0.13<br>[0.008 to 0.252]	0.109<br>[-0.017 to 0.232]	0.02<br>[-0.095 to 0.148]	.786

Abbreviations: CI, confidence interval; PFS, progression-free survival; YI, Youden Index.

DS Table S7

OS *v* PFS: Comparison of sensitivity, specificity and YI of optimized diametric threshold predicting death before 26.4 months and optimized diametric threshold predicting progressive disease before 10.8 months after therapy start. 95%-CI and *P* were obtained from bootstrapping 1000 times.

End point<br>Measurement<br>Method<br>threshold	OS<br>diameter<br>optimized<br>21.4	PFS<br>diameter<br>optimized<br>33.2	Difference	*P*
sensitivity<br>[95%-CI]	0.621<br>[0.512 to 0.714]	0.709<br>[0.623 to 0.792]	-0.088<br>[-0.204 to -0.011]	.094
specificity<br>[95%-CI]	0.677<br>[0.578 to 0.753]	0.421<br>[0.333 to 0.509]	0.257<br>[0.154 to 0.342]	< .001
YI<br>[95%-CI]	0.298<br>[0.134 to 0.415]	0.13<br>[0.008 to 0.252]	0.168<br>[-0.024 to 0.294]	.056

Abbreviations: CI, confidence interval; OS, overall survival; PFS, progression-free survival; YI, Youden Index.

DS Table S8

OS v PFS: Comparison of sensitivity, specificity and YI of optimized volumetric threshold predicting death before 26.4 months and optimized volumetric threshold predicting progressive disease before 10.8 months after therapy start. 95%-CI and *P* were obtained from bootstrapping 1000 times.

End point<br>Measurement<br>Method<br>threshold	OS<br>volume<br>optimized<br>45.4	PFS<br>volume<br>optimized<br>45.3	Difference	*P*
sensitivity<br>[95%-CI]	0.564<br>[0.459 to 0.654]	0.406<br>[0.314 to 0.496]	0.158<br>[0.078 to 0.223]	< .001
specificity<br>[95%-CI]	0.778<br>[0.688 to 0.843]	0.703<br>[0.617 to 0.787]	0.074<br>[0.005 to 0.13]	.021
YI<br>[95%-CI]	0.342<br>[0.198 to 0.449]	0.109<br>[-0.017 to 0.232]	0.233<br>[0.09 to 0.344]	.002

Abbreviations: CI, confidence interval; OS, overall survival; PFS, progression-free survival; YI, Youden Index.

DS Table S9

Diametric *v* volumetric ETS: Comparison of sensitivity, specificity and YI of established diametric threshold predicting death before 26.4 months and proposed volumetric threshold predicting death before 26.4 months after therapy start. 95%-CI and *P* were obtained from bootstrapping 1000 times.

End point<br>Measurement<br>Method<br>threshold	OS<br>diameter<br>established<br>20	OS<br>volume<br>proposed<br>45	Difference	*P*
sensitivity<br>[95%-CI]	0.561<br>[0.456 to 0.647]	0.564<br>[0.451 to 0.645]	-0.003<br>[-0.045 to 0.056]	.887
specificity<br>[95%-CI]	0.713<br>[0.622 to 0.789]	0.778<br>[0.688 to 0.844]	-0.065<br>[-0.139 to 0.013]	.097
YI<br>[95%-CI]	0.274<br>[0.129 to 0.398]	0.342<br>[0.19 to 0.444]	-0.068<br>[-0.149 to 0.038]	.143

Abbreviations: CI, confidence interval; OS, overall survival; YI, Youden Index.

DS Table S10

Diametric *v* volumetric ETS: Comparison of sensitivity, specificity and YI of established diametric threshold predicting progressive disease before 10.8 months and proposed volumetric threshold predicting progressive disease before 10.8 months after therapy start. 95%-CI and *P* were obtained from bootstrapping 1000 times.

End point<br>Measurement<br>Method<br>threshold	PFS<br>diameter<br>established<br>20	PFS<br>volume<br>proposed<br>45	Difference	*P*
sensitivity<br>[95%-CI]	0.422<br>[0.339 to 0.516]	0.389<br>[0.304 to 0.489]	0.032<br>[-0.028 to 0.098]	.325
specificity<br>[95%-CI]	0.66<br>[0.573 to 0.751]	0.703<br>[0.617 to 0.788]	-0.043<br>[-0.114 to 0.032]	.236
YI<br>[95%-CI]	0.082<br>[-0.035 to 0.207]	0.093<br>[-0.024 to 0.222]	-0.011<br>[-0.105 to 0.087]	.826

Abbreviations: CI, confidence interval; PFS, progression-free survival; YI, Youden Index.
